# Short-Term Memory Maintenance of Object Locations during Active Navigation: Which Working Memory Subsystem Is Essential?

**DOI:** 10.1371/journal.pone.0019707

**Published:** 2011-05-24

**Authors:** Oliver Baumann, Ashley J. Skilleter, Jason B. Mattingley

**Affiliations:** Queensland Brain Institute and School of Psychology, The University of Queensland, St Lucia, Queensland, Australia; University of Leuven, Belgium

## Abstract

The goal of the present study was to examine the extent to which working memory supports the maintenance of object locations during active spatial navigation. Participants were required to navigate a virtual environment and to encode the location of a target object. In the subsequent maintenance period they performed one of three secondary tasks that were designed to selectively load visual, verbal or spatial working memory subsystems. Thereafter participants re-entered the environment and navigated back to the remembered location of the target. We found that while navigation performance in participants with high navigational ability was impaired only by the spatial secondary task, navigation performance in participants with poor navigational ability was impaired equally by spatial and verbal secondary tasks. The visual secondary task had no effect on navigation performance. Our results extend current knowledge by showing that the differential engagement of working memory subsystems is determined by navigational ability.

## Introduction

As people navigate they acquire knowledge about their environment, including the spatial layout of salient landmarks, based upon visual, proprioceptive and kinaesthetic inputs. This information is encoded and stored in memory, allowing us to find our way back to a desired location within the same environment. In recent years, several studies have investigated the behavioural and neural correlates of human navigation [1]. A key question has been to understand the nature and properties of the memory systems that underlie our ability to encode and retrieve target locations in a three-dimensional environment [2], [3]. Spatial navigation is a complex mental task that depends critically on the efficient storage and updating of information, functions that are generally associated with working memory. However, the causal role of working memory in the storage of object locations during active spatial navigation has rarely been explored. A second key question is whether people adopt distinctive strategies during the encoding of novel environments, which might lead to notable individual differences in navigational ability [2], [4]. The overarching aims of the current study were to determine the contributions of distinct working memory subsystems to the maintenance of landmark locations within a novel environment, and to examine whether good and poor navigators differ with respect to their reliance on these specific working memory processes.

### The working memory model

The classic working memory model originally proposed by Baddeley and Hitch [5] is comprised of three interacting components: an *executive controller*, called the ‘central executive’, and two subservient systems: the *phonological loop* and the *visuo-spatial sketchpad*. The phonological loop is described as a store that retains verbal information for a brief period of time. The visuo-spatial sketchpad, on the other hand, is assumed to be a short-term store for both spatial and visual information. Logie [6] proposed that the visuo-spatial sketchpad be divided into separate visual and spatial subsystems, sometimes referred to as the ‘*what’* and ‘*where’* subsystems, respectively. The visual component, called the ‘visual cache’, is thought to be responsible for retaining basic visual information about the shape and colour of objects. By contrast, the spatial component, called the ‘inner scribe’, is assumed to hold information concerning the location and movement of objects, and to involve spatially based rehearsal [7], [8]. To assess the relative contributions of visual, verbal, and spatial working memory to particular cognitive operations, investigators have used dual-task paradigms in which the dependent variable is the change in primary task performance when a secondary task is undertaken concurrently during the encoding, maintenance or retention of novel information. It is this dual-task approach that we adopted in the current investigation of navigational ability.

### Working memory in spatial navigation

Taking Logie's model of working memory as a starting point, we asked which subsystem – visual, verbal or spatial – is most important for object-location memory in active spatial navigation tasks. If landmarks are represented as two-dimensional visual images, resembling distinct ‘snapshots’ of the environment [9], or as a map [10], then navigation should be most disrupted by a secondary visual task that taxes the ‘visual cache’ [6]. Alternatively, if during navigation people encode the locations of objects verbally (e.g., “object A is to the north of landmark B”; [11], [12]), then navigation performance should be most disrupted by a verbal secondary task. A third possibility is that objects and landmarks are encoded as ‘amodal’ spatial representations, that is, as representations of the geometric layout of an environment that are not exclusively within any particular sensory modality [13], [14]. If this is the case, navigation performance should be most disrupted by a secondary task that requires explicit object-location judgements, regardless of the sensory modality in which the primary and secondary tasks are performed.

To date, the contributions of the different working memory subsystems in the maintenance of object locations during active navigation remain unknown. On a closely related topic, however, two previous studies reported evidence to suggest a role for working memory in real world way-finding behaviour [15], [16]. Both studies consisted of an initial learning stage, in which participants performed a secondary task while being led through the main streets of a real town [15] or a virtual city [16]. In a subsequent way-finding phase, participants had to retrace the route as closely as possible and to stop at a specified end point. The studies by Garden et al. [15] and Meilinger et al. [16] found that when either a verbal or spatial secondary task was performed during the learning phase, it interfered with subsequent way-finding, suggesting that both these working memory subsystems are important for encoding of navigationally relevant information. However, both studies used complex spatial layouts – real or virtual – to test participants' reliance on specific working memory subsystems for way-finding behaviour. Although such an approach has the advantage of high external validity, it also complicates the interpretation of the role of working memory in spatial navigation. For example, in realistic environments the number, locations and relative salience of paths and landmarks cannot readily be quantified or controlled, which in turn makes it difficult to limit or control the types of strategies participants might employ to navigate.

Given the number of possible cognitive strategies that can be employed to encode landmark information, it is not surprising that humans differ widely in their navigational abilities [1]. When humans acquire spatial knowledge from direct experience in an environment or from media such as virtual environments or maps, individual differences are large and robust (e.g. [17], [18]). A recent study by Baumann and colleagues [2] used functional imaging to investigate the effects of navigational ability on the processes underlying object-location memory during active navigation. Dividing participants into good and poor navigators, based upon average behavioural performance, revealed that good navigators showed significantly stronger memory-related activation in striatal brain regions, whereas poor navigators showed significantly higher activity in the left hippocampus. Given the known role of the striatum in implicit learning (e.g. [19]), the findings of Baumann and colleagues [2] suggest that stronger striatal activity in good navigators might reflect a non-verbal component of the memory process. By contrast, the stronger left hippocampal activity in poor navigators is consistent with the proposal that this group of participants uses a predominantly verbal code to remember the target object's location. Due to the correlational nature of functional imaging findings, however, the study by Baumann and colleagues [2] cannot unequivocally answer the question of which memory subsystem is most essential for object-location memory during active navigation, or whether good and poor navigators differ in their reliance on these subsystems.

### The present study

The goal of the present study was to examine whether specific working memory subsystems are involved in the storage of object locations acquired during active spatial navigation. We employed a sparse virtual environment that consisted of an infinite, textured plane with three cylindrical landmarks and a distinctive, pyramid-shaped target object. We employed such an uncluttered environment to control the number, locations and relative salience of paths and landmarks. To investigate individual differences in performance, we also divided participants into ‘good’ and ‘poor’ navigators, to determine whether the degree to which participants rely on spatial, verbal or visual working memory subsystems depends on their general navigational ability. In contrast to previous studies, we investigated the storage rather than the encoding of object locations. To achieve this goal, we had participants perform secondary tasks exclusively during the maintenance interval between initial encoding of a novel layout and the subsequent retrieval phase. By taking this approach we ensured that the secondary task engaged working memory only for the period during which object location information had to be maintained, and that the various perceptual and motor demands of encoding and retrieval were free from contamination by the secondary task.

In the initial encoding phase of each trial, participants were required to navigate to and encode the location of a target object. During the subsequent maintenance period, participants were asked simply to remember the object's location (control task), or they were asked to perform one of three secondary tasks. In each secondary task, participants were required to respond when the current stimulus was the same as the previously presented stimulus (i.e., a 1-back task). In the visual secondary task, we presented coloured, flickering checkerboard patterns. Participants responded when the colour of the checkerboard was the same as the one previously presented. Previous research has shown that visual dynamic noise [20] and colour flicker [21], [22] strongly disrupt visual memory. In the verbal secondary task, four meaningless, spoken syllables were presented via headphones. Participants had to press a button when they heard a syllable that was identical to the one previously presented. It has been found that immediate recall of verbal material, such as word lists, is disrupted by the presentation of irrelevant spoken material. This disruptive effect, known as the *irrelevant speech effect* because it persists when participants are free to ignore the material [23], [24], is just as disruptive for meaningless phonemes as for meaningful words [25], and thus is assumed to be phonologically based (as opposed to semantically based). In the spatial secondary task, participants heard white noise bursts over headphones. The noises originated from one of four different azimuthal locations in virtual auditory space, and participants were required to indicate when the virtual location of a sound was the same as that of the previously presented one. A similar version of this task was used by Meilinger and colleagues [16], and was found to interfere with way-finding performance in a virtual environment. After the initial encoding and maintenance periods, participants re-entered the virtual environment and were required to navigate back to the remembered location of the target, which had been removed from the display.

Previous studies have suggested that both the verbal and spatial working memory subsystems play a role in real world way-finding behaviour [15], [16]. The aim of the present study was to examine if the same subsystems also underlie the more defined and fundamental process of object-location memory in three-dimensional environments. Additionally, we explored whether the degree to which participants rely on spatial, verbal or visual working memory subsystems depends on their general navigational ability.

## Methods

### Ethics Statement

All participants in this experiment gave informed written consent prior to inclusion in the study, and were compensated monetarily for their participation. All research was conducted under the approval of The University of Queensland Ethics Committee, and thus adhered to the ethical standards outlined in the 1964 Declaration of Helsinki.

### Participants

Twelve male (11 right-handed) and 12 female (11 right-handed), healthy volunteers (mean age 23 years, SD = 3 years) with normal or corrected-to-normal vision and normal hearing participated in the study.

### Task and stimuli

**Figure 1:**
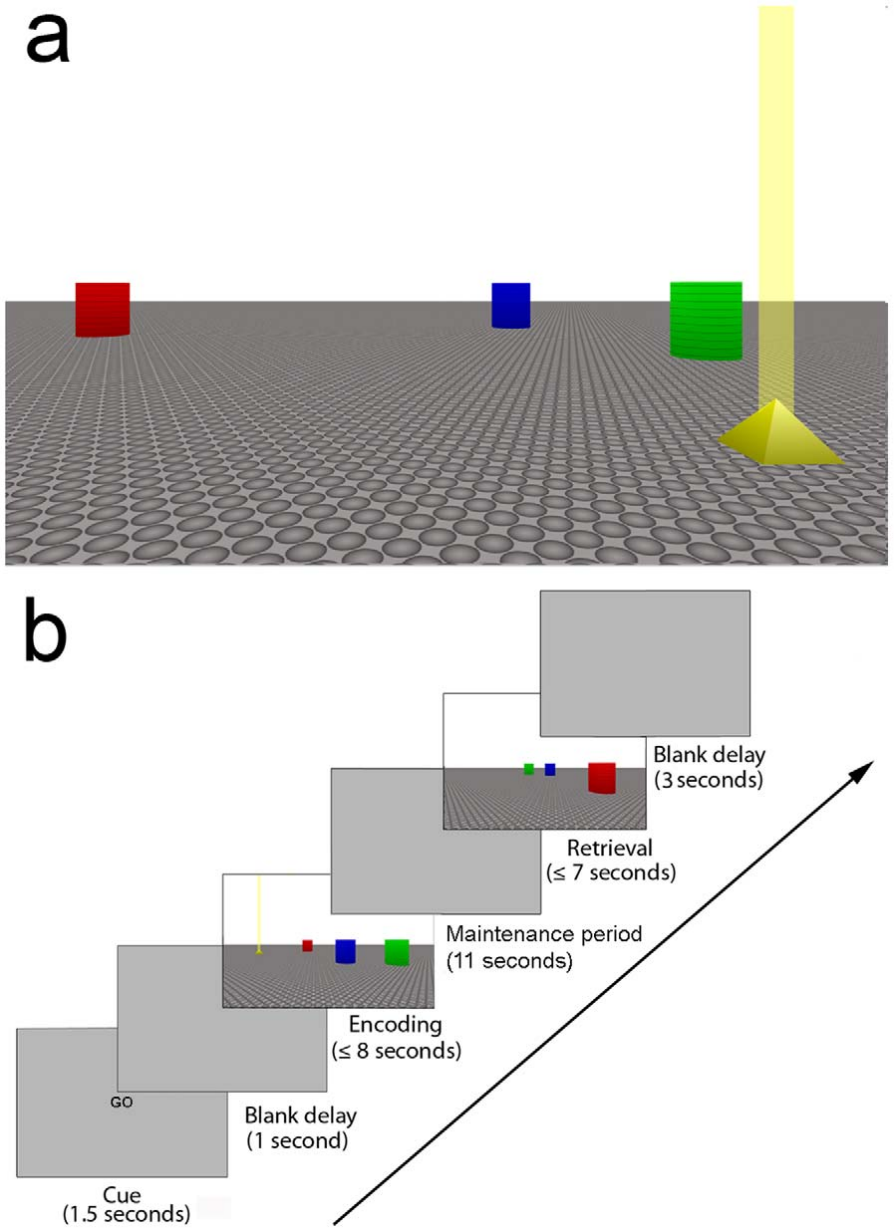
Schematic of the virtual environment used in the navigation task. (a) Example display of the virtual environment during the encoding phase of an experimental trial. Landmarks are shown in red, green and blue. The target is shown in yellow, with a virtual light beacon projecting vertically from its apex. (b) Sequence of events in a typical experimental trial. Participants entered the environment and navigated to the target before pressing a button on the joystick to indicate when they reached its location. The encoding phase was followed by a delay period (11 seconds), in which participants were asked simply to remember the object's location (control task), or they were asked to perform one of three secondary tasks (visual, verbal or spatial). In the subsequent retrieval phase, participants re-entered the arena from a different location than in the encoding phase (shifted by 90°, 180° or 270°, with equal probability). They were required to navigate to the location of the target, which was now absent from the display, and to indicate via the joystick when they had arrived there. The next trial commenced after a further delay of 3 seconds.

We used the Blender open source 3D content creation suite (The Blender Foundation, Amsterdam, The Netherlands) to create a virtual environment and administer the navigation task. Participants viewed the environment at a distance of 70 cm from a Dell 2407WFP wide-screen (52 cm ×32 cm) liquid crystal display monitor (Dell Computer Corporation, Austin, TX, USA), and moved through the virtual arena by means of a joystick held in their right hand. The arena consisted of an infinite plain with a pebble-like texture covering the ground to enhance its 3D quality. It contained four visual objects: three landmarks and one target (Figure 1a). The landmarks were cylinders (red, green and blue) with a virtual height of 2.2 meters and a diameter of 1 meter. The target was a yellow pyramid with a virtual height and width of 0.5 meters. The pyramid had a virtual ‘light beacon’, which projected vertically from the apex, to allow its position to be determined when occluded by the landmarks.

In the initial, ‘encoding’ phase participants were instructed to remember the location of the target object with respect to the landmarks, which constituted the only reliable points of reference within the virtual arena. On each trial, they were asked to navigate to the target and press a button on the joystick to indicate when they arrived there. Participants were trained to complete the encoding phase within a time limit of 8 seconds. The encoding phase was followed by a delay period (11 seconds). During this delay, participants either had no secondary task to perform (which therefore permitted active, undisrupted maintenance of the target's location), or were required to perform one of three different secondary tasks. All secondary tasks required 1-back judgements, in which participants had to respond when the current stimulus was the same as the one previously presented. Each 1-back task was composed of nine stimuli (duration 300 ms, interstimulus interval 700 ms). In the *visual secondary task*, participants were presented with coloured checkerboard patterns that were composed of one of four possible colour combinations: red-grey, green-grey, blue-grey, or yellow-grey checks, which flickered alternately at 20 Hz (check size 2.46°×2.46°; the size of the entire stimulus was 26° of visual angle horizontally and 20° of visual angle vertically at a viewing distance of 70 cm). In the *verbal secondary task*, participants were presented with four different meaningless phonemes (according to the International Phonetic Alphabet: æm, ka, tε, ku 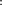) via headphones (Ear Force AK-R8, Turtle Beach, New York). In the *spatial secondary task* participants were presented with white noise bursts, which originated from four different locations in virtual auditory space (at 45°, 135°, 225° or 315° in the azimuthal plane). The sound was spatialised using Ear Force AK-R8 headphones and an Audio Advantage SRM multichannel USB Sound Card (Turtle Beach, New York), from which a virtual sound source can be accurately positioned in space. The auditory stimuli in both the verbal and the spatial task were presented at a comfortable listening level, which was the same for all participants.

In the subsequent *retrieval phase* of the navigation task, participants re-entered the arena from a location that was always different from that used in the encoding phase (shifted by 90°, 180° or 270°, with equal probability). During retrieval, the landmarks appeared in their original locations, but the target was now absent. Participants were required to navigate to the remembered location of the target and to press a button on the joystick when they arrived there. The retrieval phase had a time limit of 7 seconds. Following completion of the retrieval phase, the display remained blank for 3 seconds before participants commenced the next trial (see Figure 1b). Within the arena, the locations of the landmarks and targets were altered on every trial, requiring a completely new spatial layout be learned on each occasion. To assess whether any effects on retrieval accuracy in the navigation task might be due to baseline differences in secondary task difficulty, rather than to their unique content (visual vs. verbal vs. spatial), we also assessed participants' secondary task performance in isolation (i.e., without the primary navigation task).

The experiment consisted of a total of 60 trials of the navigation task (15 trials for each of the three different secondary task conditions, plus a further 15 trials of the control condition without the secondary task), and 45 trials of the secondary tasks on their own (15 trials for each of the three conditions) to check for any differences in overall difficulty between them. The order of presentation of the secondary task conditions was randomised. No cue was given to indicate which secondary task would appear next, to prevent participants from switching between different encoding strategies for the different secondary tasks. The experiment was divided into four blocks: an initial block of the secondary task in isolation, followed by two blocks of the navigation task, and then another block of the secondary task in isolation. Before the experimental session, participants were trained to enable them to navigate effectively within the brief encoding and retrieval periods, and to familiarise themselves with the secondary tasks. We recorded participants' absolute metric error (defined as the distance in virtual meters between the target's location and the location indicated by the participant at the completion of the retrieval interval) as a measure of their memory performance. We also recorded accuracy for the secondary tasks.

## Results

### Secondary task performance

The number of errors (defined as the sum of target omissions and false alarms) was very low for all three secondary tasks (<1% in each of the three different tasks). Chi-square tests revealed no significant difference in mean error frequency between the three conditions (Chi-square  = 1.2; df  = 2; p = 0.550). When participants performed the secondary task in isolation (i.e., without the primary navigation task) the mean number of errors was also very low (<1% in each of the three different tasks). Chi-square tests again revealed no significant difference in average error frequency between the three conditions (Chi-square  = 0.72; df  = 2; p = 0.698). There was also no significant difference in error frequency between the secondary task performed during the maintenance period of the navigation task (averaged over visual, verbal and spatial conditions) and the secondary task performed in isolation (Chi-square  = 0.23; df  = 1; p = 0.628). These results indicate that difficulty was well matched across the secondary task conditions, and that the navigation task itself did not have any measurable effect on secondary task performance.

### Primary task performance

Participants' accuracy in the navigation task was measured in terms of absolute metric error (i.e., the distance in virtual meters between the target's location and the location indicated by the participant at the end of the retrieval interval). We divided participants into ‘good’ and ‘poor’ navigators, based upon their absolute metric error, to determine whether there was a relationship between navigation ability and the effects of a secondary task during the maintenance interval. We checked whether the error distribution for the group as a whole departed significantly from normality. The metric error distribution of the control condition for all participants had a skewness of 0.264 (SE = 0.472) and a kurtosis of 1.197 (SE = 0.918), showing no evidence of non-normality (Shapiro-Wilk test p = 0.427). Following the approach of Baumann and colleagues [2], we used a median split to divide participants into two groups based on their absolute error in the control (single-task) condition (i.e., navigation performed in isolation, without a secondary task). This yielded a baseline average error for the good navigators (N = 12; 5 males, 7 females) of 1.86 virtual meters (SE = 0.14), and an average error for the poor navigators (N = 12; 7 males, 5 females) of 2.89 virtual meters (SE = 0.17).

**Figure 2:**
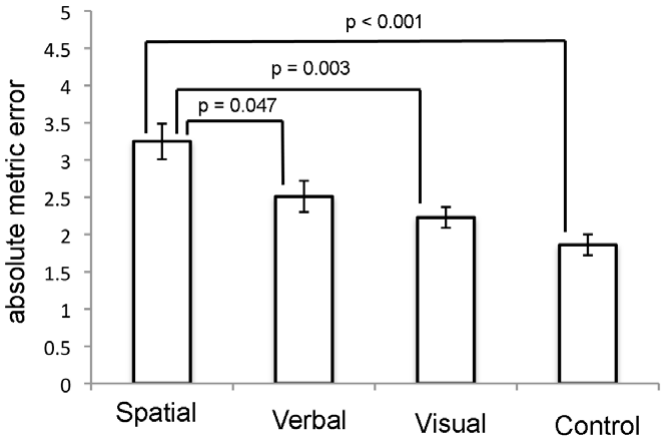
Mean absolute metric error (±1 standard error) for the good navigators plotted separately for the three different secondary task conditions and the control condition (without interference).

**Figure 3:**
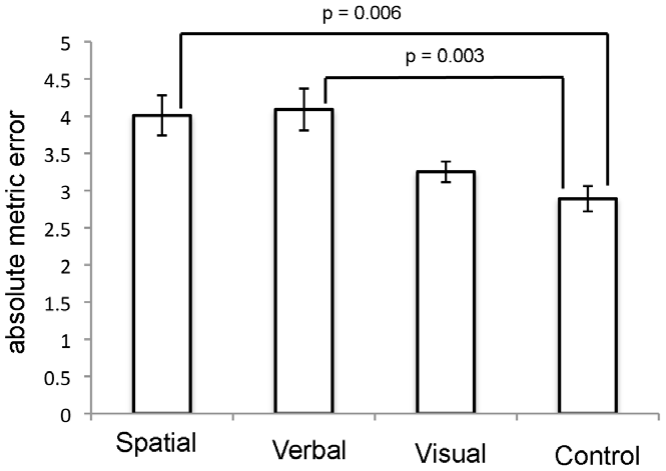
Mean absolute metric error (±1 standard error) for the poor navigators, plotted separately for the three different secondary task conditions and the control condition (without interference).

As shown in Figures 2 and 3, the effects of the three secondary tasks were clearly different for the good and poor navigators. Good navigators were more strongly disrupted by the spatial secondary task compared with the verbal and visual secondary tasks. By contrast, poor navigators showed reduced performance in both the spatial and verbal secondary tasks compared with the control condition. These trends were confirmed statistically. A repeated-measures ANOVA on navigational accuracy with the factors of Group (good versus poor) and Secondary Task (spatial, verbal, visual, baseline) revealed a significant main effect of Secondary Task (F(3,66)  = 38.821; p<0.001) and a significant interaction of Group and Secondary Task (F(3,66)  = 3.704; p<0.016). We then conducted one-way ANOVAs on absolute error for the two groups separately, to compare the effects of the different secondary tasks and the single-task baseline. These analyses revealed significant main effects for both the good navigators (F(3,44)  = 9.664; p<0.001) and poor navigators (F(3,44)  = 6.853; p<0.001)^1^ As shown in Figures 2 and 3, post-hoc Bonferroni tests conducted on data from the good navigators revealed that the secondary spatial task produced a significant impairment in navigation performance relative to the control task, but the secondary verbal and visual tasks did not. Also, the impairment produced by the secondary spatial task was significantly larger than that caused by the secondary verbal and visual tasks. In comparison, the poor navigators were significantly impaired by the spatial and verbal secondary tasks, but not by the visual secondary task. Furthermore there was no significant difference between the impairments caused by the secondary spatial, verbal and visual tasks.

## Discussion

The present study examined the relative contributions of distinct working memory subsystems on object-location memory during a landmark-based spatial navigation task. We divided participants into ‘good’ and ‘poor’ navigators, based on performance in the control (single-task) trials, because we predicted that the degree to which people rely on spatial, verbal or visual working memory might depend on an individual's general navigational ability. We employed verbal, visual and spatial secondary tasks to selectively load each of the three distinct working memory subsystems: the phonological store, the visual cache, and the inner scribe [5], [26]. We purposely chose easy (i.e. 1-back) secondary tasks, because we wanted to identify any unique contributions from visual, verbal and spatial processes, but did not want to draw heavily on general cognitive resources such as those of the central executive [27], [28]. The error rates for all three secondary tasks were statistically indistinguishable from one another, and were extremely low overall. This result suggests that the differences in performance on the navigation task were mainly due to the unique content of the secondary tasks, rather than to baseline differences in task difficulty. We found that while navigation performance in the good navigators was only significantly impaired by the spatial secondary task, navigation performance in the poor navigators was equally impaired by the spatial *and* the verbal secondary tasks. The visual secondary task did not decrease navigation performance significantly in either of the two groups of participants. These results suggest that good navigators depend strongly on a non-verbal, spatial short-term memory process during active navigation. The results also point toward reliance by this group on metric spatial features, such as the geometry of the environment. By contrast, poor navigators appear to rely heavily on verbal coding of the environment (e.g. the colour of the landmarks and their spatial configuration), which does not capture the fine-grained metric relationships between landmarks and the target object in our task.

The experimental design of our study differed in three important aspects from related studies that investigated the role of working memory in way-finding behaviour. First, in the studies of Garden et al. [15] and Meilinger et al. [16], participants had to perform a secondary task while they were encoding a novel environment. Since these learning periods were temporally extensive (in the range of minutes), the participants were not only using this period to encode the environment, but were also using it to maintain and potentially rehearse already acquired information at the same time. Based solely on the results of these previous studies it is impossible to determine if the secondary tasks interfered with the encoding process, the maintenance process, or both. In our study, the secondary tasks were only performed during the retention interval between encoding and retrieval. Therefore, all observed differences in navigational accuracy can reliably be attributed to interference with the maintenance of the distinct short-term memory components, and we are able to rule out the possibility that impairments in navigational performance could also be caused by interference with perceptual, encoding, retrieval or even motor processes. Second, previous studies used realistic spatial layouts to test participants' reliance on specific working memory subsystems for successful way-finding. Although such an approach has the advantage of high external validity, it also complicates the interpretation of the role of working memory in the most basic aspects of spatial navigation. We used a sparse virtual environment to control the number of landmarks, as well as their locations and relative salience. This in turn might also have helped limit the types of strategies participants could employ to navigate. Finally, the secondary tasks employed by Garden et al. [15] and Meilinger et al. [16] were rather heterogeneous in nature: Garden and colleagues [15] employed an articulatory suppression task and a spatial tapping task, whereas Meilinger and colleagues [16] employed a lexical decision task, a sound localisation task and a visual mental imagery task. We, on the other hand, always used the same cognitive task (i.e., the 1-back task), while varying only the nature of the stimuli. We did this to ensure that differences in performance on the navigation task could be attributed to the content of the secondary tasks, rather than to changes in the demands upon executive control.

In the study by Meilinger and colleagues [16], verbal and spatial secondary tasks were found to interfere equally with way-finding (with a trend for stronger interference caused by the verbal task). Based on these results, Meilinger and colleagues [16] proposed that humans might navigate using a spatial representation of their environment, but, in contrast to other non-human animals, they might also recode this spatial representation into a verbal format. Our study extends this notion by showing that whether humans encode environmental features in a verbal format in addition to a spatial format depends on their underlying navigational ability. We found that good navigators have a strong tendency to rely exclusively on spatial working memory, whereas poor navigators are more likely to employ a dual verbal-spatial code as proposed by Meilinger and colleagues [16]. Additionally, one might conjecture that visually rich environments lend themselves to storage in a symbolic or verbal format (as in the study of Meilinger and colleagues [16]), whereas sparse environments lend themselves to storage in a spatial, potentially more action-oriented, format. This begs the question of whether individuals with better navigational ability actively refrain from relying on a verbal code in environments that do not readily lend themselves to verbal labelling and, if so, whether it is possible to improve poor navigators' performance by training them to adopt a spatial memory strategy. Alternatively, it might be that navigational skill is a ‘hardwired’ cognitive module that cannot be modified with training.

A potential limitation of our study is that it employed a desktop virtual environment rather than a real-world environment, so that participants could not utilise internally generated movement information, such as vestibular signals, afferent proprioceptive signals, or efference copies of the commands issued to the musculature. The absence of these so-called ‘ideothetic’ cues might have affected the participants' choice of strategies and therefore contributed to the observed individual differences in performance. Future studies might usefully employ a physical version of our virtual environment to exploit the benefits of a highly controlled stimulus array while allowing participants to draw upon ideothetic cues to aid their performance.

In conclusion, our study shows that in sparse visual environments, humans with good navigational ability rely exclusively upon spatial working memory to remember the locations of landmarks and objects, whereas poor navigators rely on a combination of both spatial *and* verbal working memory. This suggests that the spatial working memory subcomponent is most fundamental for successful navigation through three-dimensional space in its most basic form. The use of an additional verbal code might be useful in environments with rich landmark detail, but in sparse and undifferentiated environments it appears to have the potential to compromise successful way-finding. Our results extend current knowledge by showing that the differential engagement of working memory subsystems is determined by navigational ability.
